# Nanotechnology in Food and Plant Science: Challenges and Future Prospects

**DOI:** 10.3390/plants12132565

**Published:** 2023-07-06

**Authors:** Mohammad Azam Ansari

**Affiliations:** Department of Epidemic Disease Research, Institute for Research and Medical Consultations (IRMC), Imam Abdulrahman Bin Faisal University, P.O. Box 1982, Dammam 31441, Saudi Arabia; maansari@iau.edu.sa

**Keywords:** plant pathogens, stress tolerance, crop growth and yield, nanosensor, food packaging, nanocarrier, genetic modification, food-borne pathogen

## Abstract

Globally, food safety and security are receiving a lot of attention to ensure a steady supply of nutrient-rich and safe food. Nanotechnology is used in a wide range of technical processes, including the development of new materials and the enhancement of food safety and security. Nanomaterials are used to improve the protective effects of food and help detect microbial contamination, hazardous chemicals, and pesticides. Nanosensors are used to detect pathogens and allergens in food. Food processing is enhanced further by nanocapsulation, which allows for the delivery of bioactive compounds, increases food bioavailability, and extends food shelf life. Various forms of nanomaterials have been developed to improve food safety and enhance agricultural productivity, including nanometals, nanorods, nanofilms, nanotubes, nanofibers, nanolayers, and nanosheets. Such materials are used for developing nanofertilizers, nanopesticides, and nanomaterials to induce plant growth, genome modification, and transgene expression in plants. Nanomaterials have antimicrobial properties, promote plants’ innate immunity, and act as delivery agents for active ingredients. Nanocomposites offer good acid-resistance capabilities, effective recyclability, significant thermostability, and enhanced storage stability. Nanomaterials have been extensively used for the targeted delivery and release of genes and proteins into plant cells. In this review article, we discuss the role of nanotechnology in food safety and security. Furthermore, we include a partial literature survey on the use of nanotechnology in food packaging, food safety, food preservation using smart nanocarriers, the detection of food-borne pathogens and allergens using nanosensors, and crop growth and yield improvement; however, extensive research on nanotechnology is warranted.

## 1. Introduction

The economic strength of a country mainly depends on agriculture and its products. Improved agricultural practices, including soil preparation and irrigation, are necessary to achieve self-sufficiency in food production and to strengthen a country. Self-sufficiency may be gained by solving agricultural problems, protecting food, and improving farmers’ knowledge about using modern agricultural technologies [1]. Globally, food safety and security are currently receiving a great deal of attention with the aim of ensuring a sustainable supply of nutrient-rich and safe food. Specifically, the Kingdom of Saudi Arabia is among the largest countries, with approximately 30 million citizens; it has been reported that about 80% of its food demand is met via importing food from other countries, which clearly poses a significant challenge to the economy, food safety, and public health [2]. Moreover, food can also be spoiled by various foodborne pathogens or chemical contaminants, which may cause diseases and sometimes the death of consumers. Thus, to ensure food safety, food handling, distribution, storage, and food quality should be improved; such measures would not only enhance food consumption but also prevent negative effects on consumers’ health [3].

Recently, nanotechnology has been used for a broad range of technical processes, including the characterization, fabrication, and regulation of various materials, in order to develop new materials and to create chemical, biological, biomedical, pharmaceutical, agricultural, electronic, and bioengineering applications [4,5,6,7,8,9,10,11,12,13]. Nanotechnology deals with the manipulation of materials (molecules or macromolecules), devices, or systems at the nanometer scale in order to create and utilize materials with unique properties. The developed nanomaterials are 1–100 nm in size and possess a high surface-to-volume ratio; they have unique physiochemical properties, including color, strength, diffusion, and toxicity [14]. Due to these unique properties, nanomaterials are also used to improve the protective qualities of food. Additionally, in the detection of microbial contamination, hazardous chemicals, and pesticides, various nanosensors and nanopackaging materials have been used to increase sensitivity and specificity. Food processing is further improved by nanocapsulation, which enables the delivery of bioactive compounds, increases the bioavailability of food, and improves the shelf life of foodstuffs [15]. Various forms of nanomaterials have been developed to improve food safety and enhance agricultural productivity, including nanorods, nanofilms, nanotubes, nanofibers, nanolayers, and nanosheets. In this study, we analyzed and highlighted the importance of the application of nanotechnology in improving food safety, security, processing, and packaging; we also address its relation to food insecurity issues, whereby nanotechnology is used to solve agricultural problems, such as post-harvest losses and crop growth and yields. Furthermore, we discuss the application of nanotechnology in biotic/abiotic stress tolerance and the detection of food allergens and food-borne pathogens using nanobiosensors to improve food productivity and quality. Additionally, we discuss the limitations, rules and regulations, challenges, and future prospects of using nanotechnology in the food industry and related industries.

## 2. Food Security and Safety

Food security refers to all people globally having physical and economic access to sufficient, safe, and nutritious food that meets their dietary needs, allowing them to live active and healthy lives. A lack of food security leads to chronic diseases due to malnutrition. The four most important pillars of food security are food availability, food stability, food utilization, and food access [16,17]. According to the United Nations Food and Agriculture Organization (UNFAO), the foremost universal challenge that is likely to appear by 2050 is an increase in food demand, ranging from 59 to 98%, due to the rapidly rising global population, particularly in developing countries, which is expected to reach 9 billion [18]. To ensure food security, a 70% increase in food production is required, which will be affected by climate change, land availability, agricultural yields, and food supply. In addition, the UNFAO noted that, while food waste occurs in rich countries due to packaging expiration and aesthetic concerns, it occurs more frequently in developing countries due to spoiling, contamination, and poor food quality [19,20]. Abiotic stresses (such as the salinization of the soil, elevated CO_2_ levels and temperatures, drought, and nutritional imbalances) have been shown to negatively affect the development and growth of plants and their productivity and quality. In certain cases, these factors have even led to the local extinction of some species. Moreover, abiotic stresses have reportedly caused an average yield loss of more than 50% in the majority of crops [21,22]. Therefore, to reduce the agricultural chemical footprint, it is essential to enhance plants’ growth and tolerance to abiotic and/or biotic stresses, agricultural productivity, and, most importantly, their quality through the application of nanotechnology and bioactive compounds found in seaweeds/plants [21]. Food waste also affects the environment by impacting land and water availability for food production and increasing greenhouse gases that lead to climate change [23].

Food safety is another major public health concern related to protecting food from physical, chemical, and biological contaminants during food preparation, processing, handling, and distribution, thereby protecting consumers [24,25]. Food safety is significantly affected by changes in food recipes, food habits, toxins, pathogens, and contaminants, which cause serious threats to people’s health [24]. In a country like Saudi Arabia, there is limited agricultural productivity and, thereby, completely relies on food imports and observance of Islamic laws; thus, food security is highly challenging for national control and regulatory institutions [26]. Farmers throughout the world are looking for innovations and new technologies that help to preserve food safety and security by enhancing crop production, soil improvement, food packaging, distribution, and food quality [27]. Conventional methods for the analysis of contaminations, pathogen detection, and improving food security are time consuming and expensive. Thus, recently, in the developing world, the use of many nanoscale materials and their devices has been developed with low-cost and large- areas for improving agricultural efficiency, soil improvement, food processing and packaging, food shelf-life, and the analysis of pathogens and their toxins [23]. 

## 3. Nanotechnology in Food Security and Safety

**Figure 1:**
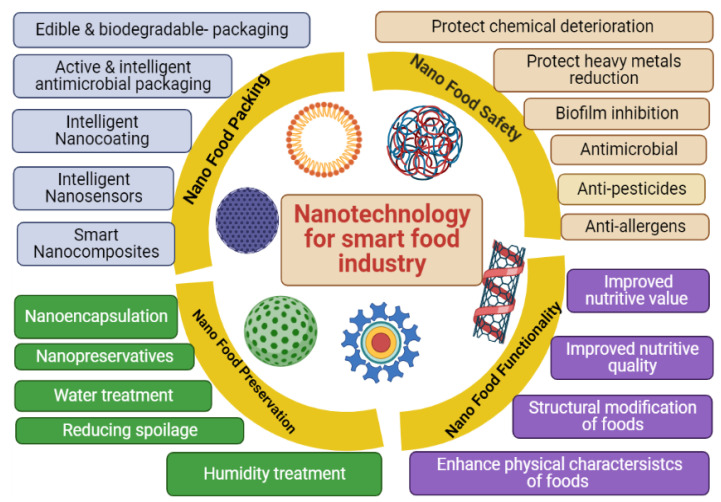
Application of nanotechnology in different areas of the food industry.

A technology that increases production while decreasing food waste is required to ensure global food security. Recently, nanotechnology has provided a path for increasing food availability and developing new products that are beneficial to agriculture, particularly by minimizing nutrient losses in fertilizers and increasing food yield through pest and nutrient management, among other things [28]. It can also be used to improve food processing, create novel food products with enhanced tastes and flavors, extend the shelf life of food products, and develop nano-based methods for detecting and preventing food spoilage (Figure 1) [29]. 

### 3.1. Nanotechnology Application in Crop Growth and Yield

Nanotechnology is used to address various agricultural issues by developing nanofertilizers, nanopesticides, and nanomaterials to induce plant growth, genome modification, and transgene expression in plants [30,31]. In a study, a chitosan nanofertilizer containing copper and salicylic acid was developed and tested for its source activity in maize plants. Seed treatment with chitosan nanofertilizer showed a significant increase in seedling vigor index and increased reserve food mobilizing enzyme activities, while foliar application suggested that used for seed treatment and foliar application of chitosan nanofertilizer significantly increased antioxidant enzymes, decreased lipid peroxidation, stimulated chlorophyll content, and ultimately suggested that nanofertilizer, by slowly releasing copper and salicylic acid, effectively enhanced crop yield by promoting source activity [32]. El-Naggar et al. [33] developed silica NPs (SiO_2_-NPs) and applied them as nanofertilizers and pesticides to maize plants. Based on the plant height, chlorophyll content, and protein composition, the data showed that minerals (nitrogen, phosphorus, and potassium) interacted with nanoparticles and could be used as growth promoters and pesticides for crops during storage at a very low and safe dose. The nanopowder waste-derived NPK fertilizers were produced from squid pin waste, shrimp shells, bovine bone, banana peels, and Kolakhar, and foliarly applied *Capsicum annum* L. cv. *Cordoba* plants were equated with commercial chemical fertilizers. The data showed that the nanocomposite increased plant growth and yield compared to commercial fertilizer-treated plants, suggesting that waste nano-NPK fertilizer can be used as an alternative to foliar fertilizer to increase Capsicum production [34]. Silver nanoparticles (AgNPs) at a dose of 15 mL/L increased the chlorophyll content, fruit yield, stem diameter, and flower percentages compared to Zinc NPs (ZnO NPs) at a dose of 7.5 mL/L. Additionally, the pollen size and spores were also enhanced by spraying AgNPs. Moreover, AgNPs-treated peach leaf extracts exerted antibacterial activity at a dose of 3000 µg/mL with a zone of inhibition in the range of 6–8.67 mm [35]. In a study, *Moringa oleifera* tree growth in calcareous soil was treated with vermicompost and NPK (NPs) fertilizer in combination for two seasons. The data showed that pod and seed yields were significantly increased, and the fixed oil of the tree was also improved by the combination of vermicompost and nanoparticles under calcareous soil conditions [36]. A study was conducted by applying 20–40 nm iron (III) oxide (Fe_2_O_3_) nanomaterials (NMs) in a hydroponic system to wheat plants. It has been reported that it increased plant height, chlorophyll content, root length, and iron content of wheat without any toxicity compared to 30–50 and 8–10 nm Fe_2_O_3_ NMs, ultimately suggesting that it could be an effective nanofertilizer for agricultural applications [37]. The effect of Fe_2_O_3_NPs (γ-Fe_2_O_3_-maghemite and Fe_3_O_4_-magnetite) was tested on barley plants, and it was reported that Fe_2_O_3_ NPs enhanced the germination rate, barley biomass, and pigmentation, thereby improving plant growth. It was also suggested that iron oxide nanoparticles could be used as nanofertilizers [38]. Recently, a novel phosphorus nanofertilizer was synthesized using hydroxyapatite NPs, pomegranate peel, and coffee ground extracts, and it was reported that the green synthesized nanofertilizer modulated photosynthetic potential, levels of carbohydrates, metabolites, and biocompatibility, and also decreased cytotoxic and genotoxic parameters in *Punica granatum* L. [39]. The effects of the three iron forms, namely, nano (Fe-NPs), sulfate (FeSO_4_), and chelated (Fe-chelated), were investigated by foliar spraying on Washington navel orange trees, and it was reported that the Fe-chelated and Fe nano forms improved vegetative growth, flower set, tree nutritional status, enhanced fruit production, and increased the quality of plant growth in environmentally preferred arid regions [40].

Nanotechnology is a new research arena, chiefly in using nanopesticides made up of NPs to control disease in plants, and is gaining attention nowadays. Bacterial panicle blight disease affects rice yield worldwide due to seed-borne rice pathogens like *Burkholderia glumae* and *B. gladioli*. A study was conducted on the biosynthesis of ZnO NPs from a native *Bacillus cereus* RNT6 strain against *B. glumae* and *B. gladioli*. The data revealed that NPs exerted antibacterial potential against *B. gladioli* and *B. glumae* by increasing the zone of inhibition, decreasing cell numbers, and inducing morphological damage, and were suggested as effective nanopesticides to act upon bacterial panicle blight disease [41]. The application of metal oxide NPs like CuO, ZnO, and FeO reduced bacterial wilt incidence and also decreased the rhizosphere bacterial community by affecting its composition and structure. Among all the metal oxide nanoparticles, CuO NPs effectively reduced the abundance of pathogens and suggested that metal oxide NPs can be used for controlling and preventing tomato bacterial wilt, a soil-borne disease [42]. A study has developed a nanopesticide formulation using chitosan and a botanical pesticide, PONNEEM^®^, which showed effective antifeedant activity and larvicidal potential against *Helicoverpa armigera* [43]. Green-synthesized CdS NPs were tested against malaria vectors and showed high toxicity in young mosquitoes, *Anopheles stephensi* and *A. sundaicus*, as well as impacting the acetylcholinesterase and glutathione S-transferase activities of the mud crab *Scylla serrata* [44].

Recently, nanotechnology has helped to distribute genetic materials to plants to perform genetic engineering and stabilize genetic materials for plant improvement [45]. In a study, it was reported that carbon dots (NPs) can act as carriers to deliver the plasmids into mature plants to develop transient plant transformation. A foliar application of plasmid-coated carbon dots on wheat plants is used to deliver cas9, gRNA, and GFP, suggesting that spray-on gene editing is simple, inexpensive, rapid, and transforms in plants [46]. In another study, double-stranded RNA that could induce RNA interference (RNAi) was sprayed on plants to modify traits without inducing genetic modification. For this, double hydroxide layered NPs with a diameter size of 50 nm were used to deliver dsRNA effectively into tomato pollen with an incubation of 2–4 h, which led to a decrease in the level of miRNA with early bicellular pollen 3 d post-treatment, thus ultimately suggesting that layered double hydroxide NPs could be used as a promising nonviral vector for other biomolecules into plant cells and effective delivery of dsRNA [47]. Carbon nanotubes with a single-walled chitosan-complex were developed using the penetration mechanism of a lipid exchange envelope into chloroplasts for delivering plasmid DNA of various plant species, including *Spinacia oleracea*, *Nasturtium officinale*, and *Nicotiana tabacum,* without the help of any chemical or biological methods. It acts as an effective transgene delivery tool compared to conventional delivery methods for the benefit of plant growth [48]. 

### 3.2. Application of Nanotechnology in Controlling Plant Diseases

Plant pathogenic bacteria, viruses, and fungi cause devastating diseases in hundreds of crops worldwide, leading to agricultural losses by affecting crop yield, quality, and growth. Thus, to counteract plant and post-harvest diseases, it is necessary to develop novel techniques and tools to bypass conventional agriculture practices. Climate change will affect crop production by increasing plant diseases and reducing the efficacy of disease-control approaches. Table 1 lists the roles of nanomaterials in plant diseases. However, nanotechnology, particularly the use of nanomaterials, effectively involves plant disease management using the following three mechanisms: (1) acting as antimicrobials; (2) acting as stimulants to induce the innate immunity of plants; and (3) acting as delivery agents for active ingredients, including pesticides and micronutrients [49]. Nanotechnology provides a solution for disease management by providing efficient formulations for the delivery of agrochemicals, nutrients, and growth regulators for the management of plant diseases that could be pathogens, thereby preventing crop loss [50]. To decrease phytotoxicity, nanocapsules improve herbicide application, enhance penetration through cuticles and tissues, and allow a slow and steady release of active ingredients inside the plant. NPs act like ‘magic bullets’, loaded with chemicals or nucleic acids, and target specific plant areas to release their charge [51]. Tomato bacterial wilt is one of the soil-borne bacterial infectious diseases caused by *Ralstonia solanacearum*. The structure and composition of rhizosphere bacterial colonies were reduced by synthesized metal oxide nanoparticles like CuO, ZnO, and FeO. Comparatively, CuO NPs significantly reduced pathogens and enhanced physiological parameters in tomato bacterial wilt-infected tomato plants, suggesting that metal oxide NPs can be effectively used against soil-borne bacterial pathogens [42,52]. In a study, TiO_2_, Ag, ZnO, and SiO_2_ NPs were synthesized with sizes of 25, 11, 35, and 25 nm and tested against a model plant-associated bacterium, *Pseudomonas protegens* CHA0, at 250 μg/mL. At a sublethal dose (500 ng/mL), NPs stimulated the antifungal effect of *P. protegens* by stimulating prm operon expression, which would enhance the synthesis of the antifungal molecule pyrrolnitrin [53]. Thus, NPs application could be an effective measure to excite the antifungal activity of pseudomonads and could be used as apromising disease control agent. 

Ginger essential oil is composed of monoterpenes and sesquiterpene hydrocarbons, which can effectively affect the cell structure and internal structure of pathogens and inhibit various phytopathogens. However, the solubility and stability of compounds in ginger essential oil are one of the important drawbacks, and they can be fixed by using nanoemulsions to increase the potential and delivery of essential oils against various pathogens to control plant diseases [54]. In a study, dsRNA was loaded onto layered double hydroxide (LDH) clay nanosheets, which are non-toxic, degradable, and show sustained release upon spraying on plant leaves. It remains in the plant for at least 30 days post-application and offers plant protection against viruses for about 20 days [55]. The protective effect of MgO NPs was tested against plant diseases using root dip, soil drench, and foliar methods and showed that MgO exerted a twin role by acting as a nematocidal and growth-promotion inducer at a concentration of 100 ppm by decreasing nematode fecundity, altering the size of galls, stimulating pigments like chlorophyll and carotenoids, enhancing protein and nitrogen content at the root and shoot, and inducing plant growth [56]. In a study, four types of copper NPs (types 1 to 4) were tested against *Fusicladium oleagineum* and *Colletotrichum* spp., which cause olive leaf spots in the olive plant. Comparatively, CuNP Type 1 and Type 2 effectively reduced mycelial growth, whereas Type 3 and Type 4 exerted strong protective effects against both species [57]. In another study, synthesized chitosan-coupled copper NPs were tested against damping-off fungal diseases in plant seeds. Chitosan-coupled copper NPs exert fungicide action by inhibiting mycelial growth and act as growth promoters by increasing growth-promoting activity in plants. It could be used as a safe alternative to commercially available pesticides by decreasing hazardous effects on the environment [58]. Nanocomposites were synthesized using copper oxide NPs decorated on the surface of reduced graphene oxide nanosheets to test against *Fusarium oxysporum,* which affects tomato and pepper plants. At 1 mg/L, the nanocomposite showed higher antifungal potential compared to fungicide Kocide 2000 (2.5 g/L) by increasing pits and pores on the fungal cell membranes, leading to cell death and thereby reducing the severity of Fusarium wilt and root rot disease. Ultimately, it was suggested that the synthesized nanocomposite could be an eco-friendly and economical nanopesticide for plant protection [59]. Green synthesized ZnO NPs using leaf extracts of *Withania coagulans* were tested for their antibacterial effect against the bacterial wilt pathogen in tomato plants. The combined treatment for 12 days of application time effectively suppressed *Ralstonia solanacearum* by decreasing disease severity and increasing plant growth [60]. Activation of ZnO NPs using visible light (405 nm, 18–30 J/cm^2^) inactivates the human pathogen *E. coli* and the wheat pathogen *F. oxysporum* without regrowth. The inactivation could be due to reactive oxygen species without affecting the visual quality of grains, preventing inhibition of germination rate, and enhancing grain seed growth, which would meet the increasing demand of the world population [61].

**Table 1:** Application of nanotechnology in the prevention and control of plant diseases.

S.No	Nanomaterials	Disease Causing Agent	Disease	Mechanism	Reference
1.	AgNPs	*Fusarium* spp. (*F. moniliforme* and *F. oxysporum*)	Damping-off of cotton seedlings.	Fungal growth was reduced by Ag NPs (25 to 200 ppm) interaction	[62]
2.	ZnO NPs	*Tomato mosaic virus*	Viral Infection in Tomato in Egypt	ZnO-NPs showed remarkably increased growth indices, photosynthetic attributes, and enzymatic and non-enzymatic antioxidants compared to the challenge control (interestingly, oxidative damage caused by ToMV was reduced by reducing malondialdehyde, H_2_O_2_, and O_2_ levels)	[63]
3.	*Bacillus*-mediated synthesized AgNPs	*S. viridans* and *R. solani*	Sheath blight disease	Destruction of mycelia of *R. solani* by reducing lesion length and enhancing root and shoot length in *Oryza sativa* seeds.	[64]
4.	AgNPs synthesized from peels of pomegranate and orange fruits extracts	*Alternaria solani.*	Early Blight Disease of Tomato in Saudi Arabia	Reduced the mycelial growth of A. solani compared to the crude extracts of two peels	[65]
5.	Copper NPs using extracts of eucalyptus and mint leaves.	*Colletotrichum capsici*	Anthracnose of chili	1000 ppm showed the highest mycelial inhibition of 99.78%	[66]
6.	ZnO NPs	*Fusarium oxysporum*	Wilt disease in *Solanum melongena* L.	Improved plant height and root length Increased content of chlorophyll a and chlorophyll b Enhanced antioxidant activity and isozymes compared to infected control	[67]
7.	ZnO NPs using *Trichoderma harzianum*	*Fusarium* sp., *Rhizoctonia solani* and *Macrophomina phaseolina*.	Fungal disease control in cotton	Reduced mycelial growth	[68]
8.	Chitosan/dextran NPs	*Alfalfa mosaic virus*	Viral infection in *Nicotiana glutinosa* plants	Induced resistance, enhanced carbohydrates, and phenolic contents Increased expression of peroxidase and phenylalanine ammonia-lyase	[69]
9.	Silicon dioxide NPs using saffron extract	*Rhizoctonia solani*	Damping-off and root rot diseases	Reduced mycelial radial growth Increased photosynthetic pigments (chlorophylls and carotenoids) Decreased oxidative stress by activating SOD, APX, CAT, phenolics, and flavonoids	[70]
10.	Biosynthesized selenium NPs using *B. megaterium*	*Rhizoctonia solani*	Rhizoctonia root-rot disease	Improve morphological and metabolic indicators Minimize the severity of root rot disease	[71]
11.	Gold NPs synthesized from *Tinospora cordifolia*	Pseudomonas aeruginosa	Biofilm-related infections	Reduced number of biofilm-forming cell internalization of the NPs, probably affecting the tendency of *P. aeruginosa* to colonize on the surface of the NPs	[72]

### 3.3. Application of Nanotechnology in Improving Stress Tolerance

**Figure 2:**
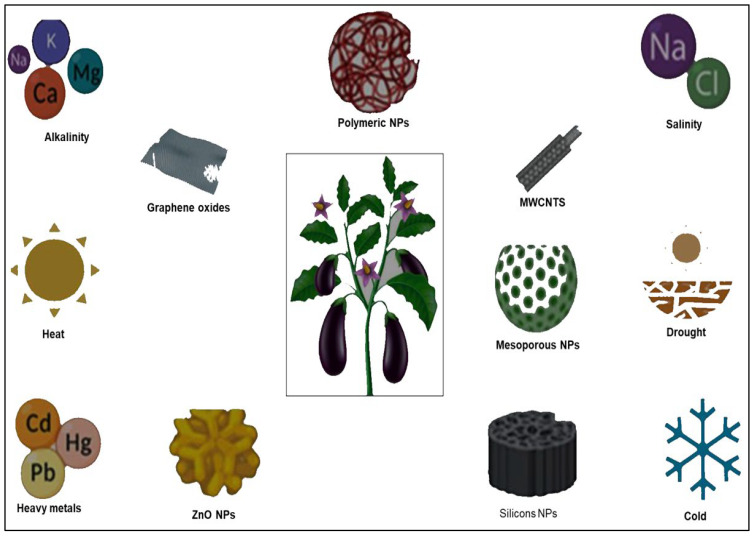
Nanotechnology in stress tolerance.

Salinity is an abiotic stress that has destructive effects on plants by affecting their growth, development, and yield, including rapeseed. Selenium and ZnO NPs were synthesized using plant hormones to study the effects of the germination process under salt stress. NPs reduced abscisic acid and regulated gibberellic acid, which promoted early seedling growth and seed germination under salt stress in rapeseed plants [73]. A carbon quantum dot nanoparticle is functionalized with proline to evaluate its effect on grapevine plants under salt stress. The NPs are reported to increase leaf fresh and dry weight; enhance superoxide dismutase, glutathione peroxidase, and catalase activities; enhance carotenoid, phenol, and anthocyanin content; and decrease malondialdehyde and hydrogen peroxide levels in plants under salinity (Figure 2). Ultimately, carbon quantum dots with chemical priming agents showed their plant priming effect by decreasing abiotic stress-related damage in plants [74]. Yttrium doping-stabilized γ-Fe_2_O_3_ NPs were supplied in nutrient solutions to *Brassica napus* plants, and it was found that NPs reduced H_2_O_2_ and lipid peroxidation, increased chlorophyll content, and improved plant growth under drought stress. Thus, maghemite NPs could be effectively used as plant fertilizer and help in drought stress management when compared to chelated iron [75]. Arbuscular mycorrhizal fungi (AMF) are symbionts that assist plants in overcoming environmental stress. Nanotechnology was used to retain AMF activity, for which TiO_2_ NPs were tested for the activity of AMF in bean roots under salinity stress, suggesting that TiO_2_ NPs showed positive effects confined to AMF under salinity stress and not on bean plants [76].

Synthesized chitosan–selenium NPs were foliar sprayed on bitter melon (*Momordica charantia*) under salinity stress, and it was reported that NPs increased antioxidant enzyme activity, proline, water, and potassium, and decreased malondialdehyde, hydrogen peroxide, and sodium aggregation in plant tissues, thereby enhancing salinity tolerance [77]. Foliar application of chitosan NPs improved banana plant growth, maintained the nutrient content of plants, increased the fresh and dry weights, stimulated photosynthetic pigments, decreased MDA, reduced the accumulation of free radical species (H_2_O_2_, ^•^OH, O_2_^•^), enhanced the accumulation of osmoprotectants (soluble carbohydrates, proline, and amino acids), and ultimately developed cold stress tolerance [78]. 

Fluoride pollution stimulated bioaccumulation in roots and shoots that inhibited reproduction, agronomic development, and uptake of minerals and decreased enzymatic antioxidants, and enhanced reactive oxygen species-mediated oxidative injury. Banerjee and Roychoudhury [79] reported that nanomaghemite decreased fluoride accumulation, stimulated the uptake of potassium, calcium, iron, copper, zinc, and selenium, and decreased the translocation of cobalt. It also triggered plant growth; stimulated antioxidant enzymes like SOD, CAT, and GPX; enhanced proline, anthocyanins, and flavonoids; and ultimately helped plants develop the potential to scavenge free radicals. Thus, it was reported that iron nanotechnology is involved in stimulating safe rice cultivation in fluoride-polluted environments. Similarly, magnetite NPs help in decreasing the salt stress effects by increasing proteins, chlorophyll content, SOD, and glutathione in two wheat cultivars (Misr1 and Gimmeza11) and reducing the lipid peroxidation marker MDA content [80]. In another study, green-synthesized gold NPs were reported to modulate the K^+^/Na^+^ ratio, chlorophyll content, defense systems, nitrogen intake and output, stomatal responses, and oxidative stress in wheat plants under high salt stress [81]. Cerium oxide NPs enhanced plant photosynthesis, modified the anatomy of plant roots, and enhanced plant salt stress tolerance by forming apoplastic barriers in brassica roots, which allows more Na^+^ transport to shoots and less accumulation of Na^+^ in plant roots [82].

### 3.4. Application of Nanotechnology in Post-Harvest Loss Reduction

Worldwide, greater than 40% of food losses, including vegetables, cereals, fruit, fish, meat, and milk products, occur due to post-harvest, food processing, trade, and customer stages in both developed and developing countries. Zeolitic imidazole framework-8 (ZIF-8) nanocomposites were synthesized with tunable pore sizes and high surface areas and functionalized with catalase by controlled self-assembly of silanes or tannic acid and Fe^3+^. Nanocomposites are reported to be an effective strategy that possesses high acid resistance capacity, effective recyclability, high thermostability, and enhanced storage stability [83]. Certain bacteria are necessary for the improvement of soil quality and the enhancement of plant nutrient acquisition. In a study, nanocoating was synthesized with N-hydroxysuccinimide-modified poly (γ-glutamic acid) and calcium ions to attach to the surface of bacteria (*P. stutzeri* NRCB010) via the formation of covalent and ionic bonds on the surface of the bacteria. Nanocoating enhanced the bacteria’s resistance to harsh environments compared to uncoated bacteria and enhanced the shelf life of bacteria [84].

A photocatalyst, TiO_2_ was reported to possess excellent bactericidal potential under UVA light. Three methods, namely, the glass cover-slip, indented coupon, and direct spreading, were used to determine the bactericidal potential of TiO_2_, and among them, indented coupon techniques were more effective in determining the bactericidal potential of TiO_2_ [85]. Hannon et al. [86] reported that copper and AgNPs with polystyrene-polyethylene oxide block copolymer (PS-b-PEO) are used for coating food packages. Comparatively, silver coating lowered the migration of nanoparticles to humans compared to copper. However, silver and copper nanoparticles are safe for use in food packaging, but the margin of exposure to humans exceeds regulatory limits, so it is suggested that they should be confined to nanopackaging applications. A nanocoating agent, chitosan NPs, was developed using sodium tripolyphosphate penta basic via the ionic gelation method to extend the mean life of fresh-cut bell peppers by preventing microbial contamination with *Listeria monocytogenes* and *Salmonella enterica*. Nanocoating protected fresh-cut bell peppers for 12 days at a temperature of 5 °C without affecting weight or sensory quality [87]. In another study, *Staphylococcus aureus*, other microflora, and *Pseudomonas fluorescens* were isolated from raw chicken and tested against low-density polyethylene films with a silver coat. Additionally, chicken breast fillets were also packed with low-density polyethylene films with a silver coat using skin packaging with a vacuum upon storage. Nanocoating extends the shelf life of chicken breast fillets apart from showing antimicrobial activity and enhanced oxidative stability; thus, it is used in several food applications such as antimicrobial packaging [88].

Nanomaterials with chitosan coating films can control microbial growth with sustained nutrients (mesophilic aerobic, yeast, and mold populations) by modulating weight loss, titratable acidity, and ripening index with a gradual increase in peroxidase enzyme and polyphenol oxidase activities to improve the shelf life of fresh blueberry [89]. Chitosan and chitosan-thyme essential oil-based nanocoatings serve as effective antibacterial agents against *Pectobacterium carotovorum*, which would cause significant economic losses to green bell peppers. The 12-day storage of nanocoated green bell pepper preserved fruit quality during ripening by lowering CO_2_ production, maintaining weight loss and firmness, decreasing respiration and ascorbic acid, and lowering colony-forming units and the incidence of disease [90]. Park et al. [91] reported that spray application of nanocoating using metal–organic complexes of tannic acid and ferric ions maintained mandarin oranges and strawberries by increasing post-harvest shelf-life, and also that spray coating was developed very rapidly in less than 5 s. Bell peppers are affected greatly due to postharvest diseases caused by the fungus Alternaria alternate. To prevent this, a study has developed chitosan NPs using -pinene (P-CSNPs) and a nanostructured edible coating on bell peppers and observed them for 21 days to analyze the quality of post-harvest. Both the nanoparticle and edible coating inhibited the growth of the fungus and preserved the physicochemical quality by maintaining weight loss and increasing the total carotene content without changing firmness, color, maturity index, antioxidant potential, or total flavonoid content during the cold storage period [92].

Chitosan NPs containing *Citrus aurantium* essential oil were developed to evaluate their protective effect on the postharvest quality of white button mushrooms. A study reported that there was a gradual release of citrus aurantium essential oil from nanoparticles, which improved the shelf life of white button mushrooms by 15 days. Additionally, NPs enhanced phenolic content, ascorbic acid, the antioxidant enzymes catalase, and superoxide dismutase, and decreased polyphenol oxidase activities at the end of the storage period [93]. Corn leaf biomass was used to synthesize edible nanocoating bioplastic for developing drug and capsule coatings. Bio-plastic nanocoating maintains tensile strength, pH, cellulose content, and ions; thus, it was suggested to be effectively used in biomedical and medical components in the pharmaceutical industries [94].

### 3.5. Nanotechnology in Genetic Modification 

In 1950, the green revolution helped in the development of high-yielding varieties of semi-dwarf wheat and rice, which showed the importance of plant breeding in a rapidly growing population. Compared to conventional plant-breeding approaches, genetic engineering methods in plant genomes can be introduced and improved in plants [95]. However, these genetic engineering tools insert genes into a random location in the plant genome and may cause undesirable outcomes. Recently, nuclease-based genome-editing methods, including TALEN (transcription activator-like effector nucleases) and CRISPR (clustered regulatory interspaced short palindromic repeats), have been used for gene targeting to efficiently introduce novel traits into the genome [96]. The plant cell wall acts as a barrier for the delivery of exogenous biomolecules into plants. More recently, for genetic engineering applications, nanomaterials have been extensively used for the targeted delivery and release of genes and proteins into plant cells. In this section, we discuss the importance of nanomaterials in plant genetic engineering. NPs of mesoporous silica act as carriers in Cre recombinase protein delivery by the biolistic method into maize plant cells. Upon release of Cre within the cell, it leads to loxP site recombination and gene elimination. A visual selection was made to opt for recombination events for the regeneration of fertile plants. Thus, it was suggested that mesoporous silica nanoparticles can be used in the accommodation of desired enzymes that influence the desired cells, and they provide an alternative tool for genome-editing technologies devoid of DNA in plants [97]. In a study, efficient diffusion-based biomolecule delivery was performed on intact plants of several species using pristine and chemically functionalized nanomaterials. Strong protein expression and efficient DNA delivery in the absence of transgene integration were facilitated with the help of nanomaterials and also protected DNA from nuclease degradation in the leaves of *Gossypium hirsutum*, *Eruca sativa*, *Nicotiana benthamiana*, and *Triticum aestivum* [98]. Chitosan-complexed single-walled carbon nanotubes offer a promising tool for effective plasmid DNA delivery to different chloroplasts of plant species, including *Spinacia oleracea*, *Nicotiana tabacum*, *Nasturtium officinale*, and *Eruca sativa* plants, and isolated *Arabidopsis thaliana* mesophyll protoplasts, without external biological or chemical aid [48,99]. Similarly, in another study, nanotubes were developed to deliver siRNA successfully, silence endogenous genes, and induce siRNA protection from nuclease degradation, thus helping in applications of plant biotechnology that focus on the delivery of RNA to intact cells [100]. Quantum dots were functionalized with β-cyclodextrin molecular baskets that enable the loading and delivery of diverse chemicals, and additionally, coating NPs with a designed guiding peptide helps in targeting their delivery specifically to chloroplasts (74.6 ± 10.8%); thus, the targeted delivery of nanomaterials with chemical cargoes guided by biorecognition motifs helps in plant genetic engineering applications [101]. Low-pressure spray application of carbon dots with a spreading surfactant for delivering small interfering RNA into the model plants *Nicotiana benthamiana* and tomato (*Solanum lycopersicum*) exhibited strong silencing of GFP transgenes that encode two subunits of magnesium chelatase, an enzyme necessary for chlorophyll synthesis in both species [102]. Yong et al. [103] reported that layered double hydroxide (LDH) NPs (40 nm) deliver dsRNA efficiently into intact plant leaf cells of *Nicotiana benthamiana* and exhibit >70% downregulation of targeted transgene mRNA within 1 day of exogenous application. A star polycation (SPc) assembles with ds-miRNA via electrostatic interaction to form a complex of nano-sized ds-miRNA/SPc that penetrates the root cortex and can be transported to vascular tissue in the seedlings of *Arabidopsis* and maize to suppress target gene expression by increasing the concentration of mature miRNA [104]. The foliar application of nanoscale copper (CuO nanosheets and commercial CuO NPs) was tested for soybean sudden death syndrome, and it was found that, comparatively, nanosheets made up of Cu showed a high level of disease suppression compared to CuO NPs because of increased leaf surface, affinity, and copper dissolution, and modulated antioxidant enzyme activity and fatty acid profile to decrease pathogenicity [105].

N-acyl-homoserine lactone lactonase from the phosphotriesterase-like lactonase family effectively inhibited plant pathogen *Erwinia amylovora* infection by degrading AHL. Moreover, the combination of directed enzyme evolution and peptide nanostructure encapsulation effectively improved the thermal resistance and shelf life of the enzyme, making it a potential candidate for antibacterial treatment [106]. In a study, different sizes (5–20 nm) and shapes (spheres and rods) of DNA-modified gold NPs (AuNPs) were investigated for their transport into *Nicotiana benthamiana* leaves. The smaller and rod-shaped AuNPs are more rapid in effectively inducing gene silencing in mature plant leaves compared to the larger and spherical AuNPs [107]. In tobacco cells, polyethyleneimine (PEI)-coated mesoporous silica NPs (positively charged) deliver GUS-encoding plasmids using ultrasound treatment with optimal conditions with varied intensities of 160, 320, and 640 W for 8 min and a size of 100 ± 8.7 nm, which is suggested as an economical method for plant cell gene transfer without any requirement for complex instrumentation [108]. Carbon nanotube (CNT) NPs efficiently deliver DNA into arugula, wheat, and cotton, which results in high protein expression levels [109]. AgNPs (100 nm) act as a gene carrier efficiently in *Nicotiana tabacum* L. compared to gold microcarriers (0.6 micron) via the biolistic transformation method under conditions of varying helium pressure (1100 psi, 900 psi, 650 psi, and 450 psi) and target distance (9 cm and 6 cm) and produced the highest number of transient GUS expression [110]. Engineered cobalt ferrite NPs (Tb, Co, and Fe) and MnFe_2_O_4_ magnetic NPs (MNPs) were hydroponically applied to barley, and it was found that NPs translocated from the roots to the leaves and increased plant growth by incorporating iron within tissues and modulating photoluminescence. NPs also increased catalase activity in leaves and altered chlorophyll content without changing carotenoid content [111,112].

### 3.6. Nanotechnology in Food Processing

To enhance nutritional properties, safety, and competence, many food industries are searching for new technologies that can increase food quality. Nanotechnology, including nano-based food additives, nanoencapsulation, and NPs, is developed for food production and processing. Food coatings with a novel biopolymer-based nanocomposite are developed that show smart and active (temperature sensing) properties by acting as temperature indicators and changing color from 8 °C to deep purple. Nanocoatings effectively enhance Ricotta cheese shelf life, and due to their temperature-sensing potential, they warn about cheese storage upon temperature alteration [113]. Nanocomposite composites have also been used as antifouling agents and can have a longer life. Nanocomposites developed by adding nanomaterials at low loadings to the main matrix showed very effective antifouling potential when compared to a virgin matrix [114]. A study was conducted to understand the polylactic acid films with migratory AgNPs into simulants of acidic food upon high-pressure food processing (200 and 400 MPa) and suggested an acceptable nanocomposite film for acidic food upon higher pressure handling processes [115]. A food-grade pickering stabilizer (size 357.8 nm) was developed using insoluble rice peptide aggregate nanoparticles via an ultrasonication process that enhanced the antioxidant and emulsifying potentials [116]. Nanofiber coating on fish fillets decreased the growth of TPB compared to that of an uncoated control. Comparatively, the textural profile (springiness and cohesiveness) of nanofiber-coated fish fillets was better at 4 °C than that of the control [117]. To enhance the shelf life of guava fruits as packs, a nanocomposite film using chitosan made up of leaf extract from Urtica dioica derived from ZnO and copper oxide was developed, which exhibited antioxidant and antimicrobial potential and thus could be a promising packaging method for improving fruit shelf life [118]. Nanomaterials help in increasing bioavailability, antimicrobial potential, improved sensory qualities, and the delivery of bioactive compounds for meat processing and packaging [119]. The nanofiltration process helps modify the daily product by removing salt from lactose, and the nanofabrication method for heat and mass transfer improves the heat resistance of packages [120]. Additionally, the nanoencapsulation process helps to regulate and release the active ingredients, protect them from chemicals, heat, and moisture, and enhance thermal stability and photostability [121,122]. A study was conducted to develop nanoparticles from enzymatically hydrolyzed soybean protein isolate to increase the production of meat-like process flavors [123]. Metal oxide NPs (SiO_2_ and TiO_2_) have been used as coloring ingredients in food substances. Additionally, to transport scents in food materials, SiO_2_ nanomaterials are used in wasted food nanomaterials [124].

### 3.7. Nanotechnology in Food Packaging

Recently, food industries have used various methods to improve food packaging to avoid food spoilage and improve its shelf life. Food packaging is necessary to protect food, fruits, and vegetables from contamination, including bacteria and the environment, to ensure their quality and safety during transportation and storage (Table 2) [125]. Nanotechnology is one of the best technologies that have been developed. It is a simple and reliable method that uses nanomaterials for food packaging to improve freshness, provide antibacterial protection, and regulate water vapor permeability. In this section, we discuss the use of nanomaterials in food packaging [126]. Interestingly, a study developed a dual-functioning 3D printed cushioning and anti-bacterial biodegradable packaging consisting of a shell (carboxymethyl nanocellulose) and chitosan or silver NPs immobilized in the core, which had an antibacterial effect on *E. coli* and *S. aureus* and had good cushioning and resilience performance [127]. TiO_2_ NPs in chitosan/tannic acid showed thermal stability, exerted antioxidant potential, and were reported to be effective for active food packaging [128]. *Nigella sativa* seed extract zinc nanostructures showed broad-spectrum quorum sensing inhibition in *P. aeruginosa* and *C. violaceum* biosensor strains and inhibited the biofilm formation of pathogens. Nanoparticles were also reported to be non-toxic and used as packaging materials for food [129]. Biosorbed-AgNPs were synthesized using plant extracts to develop biocomposite films by incorporating NPs into chitosan or PVA polymers. Nanoparticles showed antimicrobial activity by inhibiting *P. fluorescens* growth and improving tensile strength and could be used as active packaging [130]. In 3D paper tubes, ZnO NPs were developed as an absorbing pad to keep raw chicken meat underneath. It was found that absorbing pads inhibited *Campylobacter jejuni* in raw chicken meat by immobilizing zinc oxide nanoparticles with a controlled release of Zn^2+^ [131]. A nickel nanocomposite was developed using starch grafter polymers and nickel nanoparticles. After 60 days of soil burial, the breakdown of starch-grafted polymers was studied, and it was discovered that the degradation of the nanocomposite was 20.07% and that it can be effectively used as a packaging material for food items [132].

A nanohybrid system was developed using chitosan and mesoporous silica NPs with a silver nanoshell coating. Nanohybrids exerted antimicrobial activity against *S. aureus, E. coli, C. albicans*, and *A. niger* in a concentration-dependent manner (10–40 mg) and could be used effectively in food packaging and textile industries [145]. *Chloroxylon swietenia*-mediated synthesized-AgNPs inhibited food pathogens, including *Bacillus subtilis, S. gallinarum, Staphylococcus nepalensis, P. stuteria,* and *Enterococcous faecalis*, and could be used in food packaging industries worldwide [146]. An edible coating was developed using AgNPs together with calcium alginate and applied to strawberries and loquats to enhance their shelf life. The coating improved acidity loss, soluble solid content loss, and weight loss, and also enhanced the quality of the fruits [147]. Different concentrations of europium oxide NPs were doped with methyl cellulose films and tested against foodborne pathogens. Nanofilms effectively inhibited *E. coli, S. typhimurium,* and *S. aureus* and could be efficiently used in food packaging [148]. Nanocomposites of selenium and chitosan NPs synthesized using pomegranate peel extracts and *Fenneropenaeus indicus* shells effectively stimulated the lysis and deformation of *P. digitatum* hyphae within 12 h of treatment. Additionally, the nanocomposite coating of infected oranges showed decreased green mold infection signs, and it is considered an effective antifungal and edible coating to inhibit post-harvest fungal pathogens [149]. Ag- and ZnO-blended nanocomposite films effectively inhibited Gram-negative bacteria, and thus they could be an active packaging material to protect food from bacterial contamination [150]. Nanoencapsulated luteolin films consisting of a chitosan matrix showed controlled release of luteolin from the film, exerted an antioxidant activity for about 10 days, and could be used as an active packaging material [151]. Nanofibers were developed using ethylcellulose, PCL, gelatin, and *Zataria multiflora* essential oil, and ZnO NPs were used as substrates for food packaging. The developed nanofibers possessed enhanced thermal stability, the highest mechanical parameters, and antioxidant and antifungal properties against *P. notatum* and *A. niger* [152]. Active pads were developed using chitosan/cellulose nanocrystal films for chicken meat packages and it was found that nanocrystal films improved the thermal stability and oxygen barrier, increased shelf life, exerted antibacterial potential by inhibiting fungicidal, Gram-negative, and Gram-positive bacteria activity against *C. albicans*, decreased meat spoilage, and thus could act as a green alternative for food packaging [153]. Gelatin-based films were improved using thymol-loaded zein and dialdehyde kappa-carrageenan (DAK) nanoparticles. Comparatively, tensile strength antioxidant and antimicrobial activities were higher in gelatin/DAK with the blending ratio (1:2) with thymol-loaded zein NPs for application in active food packaging [154]. In a study, starch nanofilms with garlic exerted antibacterial resistance against *S. aureus* bacteria, whereas starch films with curcumin and octaphenyl-POSS showed no zone of inhibition. Comparatively, garlic-containing nanofilms did not show surface activity with stronger mechanical potentials, and this nanofilm can be used for packaging all food products, including milk products and fatty foods [155].

Nanofilms were developed using sodium alginate, cellulose nanowhiskers, and copper NPs, and were reported to inhibit microbial contamination of fresh-cut peppers via their antibacterial activity against *Trichoderma*, *E. coli*, *S. aureus*, *C. albicans*, and *Salmonella* sp., and their antioxidant potential via free radical scavenging effects. It is suggested to be an active food packaging system for its use in the food industry [156]. Naskar et al. [157] developed nanosheets using Ag-ZnO-reduced graphene oxide and polyethylene glycol (PEG), and they showed antibacterial activity against *S. aureus* and *P. aeruginosa* even after keeping them at room temperature for about 90 days. This cost-effective synthesis method was suggested to be effective in developing an active food packaging solution to overcome conventional food packaging agents. Nanofibers developed using pullulan, PVA, and thymol-loaded porphyrin metal NPs are reported to exert antibacterial activity against *S. aureus* and *E. coli* and are a good biocompatible, biodegradable, and biosafety polymeric fibers for active food packaging that would help the food industry [158]. It has been reported that palladium and platinum NPs show antibacterial effects against *E. coli*, *L. monocytogenes,* and *S. aureus* by interacting with the bacterial cell wall and inhibiting biofilm formation, with no cytotoxic effect in human renal tubular epithelial cells, human keratinocytes, human dermal fibroblasts, or human coronary artery endothelial cells [159]. A nanocomposite film was fabricated using carboxymethyl chitosan and nano MgO, which showed enhanced thermal stability, increased crystallinity, better UV-shielding, increased elasticity, ductility, and water-insoluble properties, thereby improving the use of this film on water-rich food, and also enhanced antimicrobial activity against *Shewanella baltica* and *L. monocytogenes* [160]. Biogenic silica NPs synthesized from groundnut shells were reinforced to fabricate PHBV/SiO_2_ nanocomposites, which exhibited enhanced physical, chemical, thermo-mechanical, and biodegradation properties, increased cell viability in mouse fibroblast cells, and showed antibacterial effects against *S. aureus* and *E. coli* [161]. PLA-based composite films were reinforced with acetylated cellulose nanocrystals and ZnO NPs, which improved oxygen, water vapor barrier optical and mechanical strength, UV blocking, antibacterial properties against *S. aureus* and *E. coli,* and thermal stability, and were highlighted as promising options for active food packaging materials [162]. The solution casting method was employed to develop novel active films using pullulan, carboxylated cellulose nanocrystal (C-CNC), and tea polyphenol, which showed increased water barrier properties, thermal stability, tensile strength, UV-barrier properties, antioxidant activity, and antimicrobial activity, and was suggested to be used as active food packaging [163].

### 3.8. Nanotechnology for Detection of Food-Borne Pathogens

**Figure 3:**
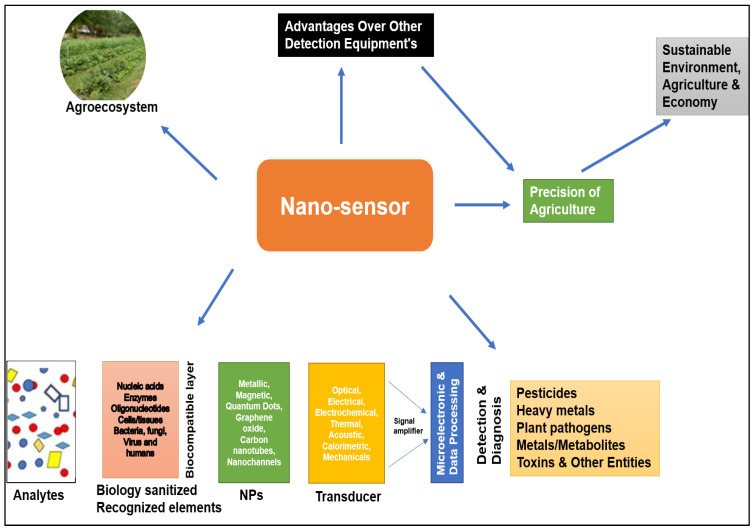
Nanotechnology-based biosensor for agriculture.

To defend against pathogens and herbivores, plants use secondary metabolites, including polyphenols. In a study, molecular sensors were developed for plant polyphenol imaging in real time via near-infrared fluorescent single-wall carbon nanotubes. Nanotube sensors showed changes in total polyphenol levels after pathogen challenge in *Soybean glycine max* and *Tococa* spp. (Figure 3). Additionally, it was found that polyphenols were released from the roots of soybean seedlings within 24 h after the pathogen attack. Thus, this sensor helps in understanding plant pathology via real-time visualization [164]. The various nanoparticles employed for the detection of foodborne pathogens are shown in Supplementary Table S1. In another study, plant pathogens were detected using a rapid and sensitive detection method, specifically a nanoparticle-based electrochemical biosensor targeting pathogen DNA via recombinase polymerase amplification, together with a gold nanoparticle-based electrochemical assessment using differential pulse voltammetry. In comparison with conventional PCR and gel electrophoresis methods, nanoparticle-based sensors exerted 10,000 times more sensitivity in identifying infected plants prior to visible disease symptoms [165]. A rice fungal disease false smut affecting rice production is caused by *Ustilaginoidea virens.* To detect false smut, a study developed a graphene-based geno-biosensor on paper electrodes as a biological recognition element used as probe DNA (cost-effective), and to enhance detection, oxidized graphene was used (up to 10 fM of target ssDNA) to determine the sensitivity of the sensor. In the presence of a pathogen, probe single-strand DNA hybridized with pathogen target ssDNA was analyzed using linear sweep voltammetry and cyclic voltammetry [166]. A multiwalled carbon nanotube-based zinc nanocomposite was used to detect the Chili leaf curl beta satellite (ChLCB). Zn-NPs are used for the immobilization of the probe ssDNA strand, and upon binding of the target DNA to ssDNA, it leads to hybridization, which was analyzed using cyclic and differential pulse voltammetry, and this nano DNA sensor was reported to be three times more specific compared to the conventional method [167]. Citrus plants are affected by the *Citrus tristeza* virus, leading to the destructive disease Tristeza. A label-free impedimetric biosensor electrodeposited with AuNPs was developed for the detection of the DNA of the *Citrus tristeza* virus. Nanosensors specifically detect target viruses in the presence of non-specific DNA with enhanced reproducibility [168]. Curl New Delhi viral DNA from tomato leaves was detected using bifunctional and monofunctional AuNP oligo probes via DNA hybridization assays, either directly or sandwiched together with silver enhancement. This method showed greater detection sensitivity (concentrations of 100 pM to 100 M) with cost-effectiveness, and it was less time consuming [169].

Zare et al. [170] developed an electrochemical biosensor label-free containing l-cysteine/PAMAM dendrimer with a gold electrode decorated to target the ITS sequence of *M. simiae*. Differential pulse voltammetry was used to measure alterations in the free guanine signal with ssDNA target concentration changes, and the biosensor showed excellent selectivity with a 1.40 fM detection limit and with great precision and high reproducibility. A study was designed to detect target DNA sequences, specifically hlyA, Bt cry1Ac, and invA genes, from *L. monocytogenes*, *B. thuringiensis*, and *Salmonella typhimurium*, and developed molecular beacons using semiconductor quantum dots for genes. With a gradual increase in the concentration from 1.17 nM to 40 nM, target molecules (in plant leaves, milk, or water) showed enhanced fluorescence at the molecular beacon in real-time monitoring, and thus the molecular beacon can be effectively used in a plant [171]. The AuNP sensor was used to detect the genomic DNA of *Klebsiella pneumoniae* and was compared to PCR. Comparatively, in the presence of target DNA, the color of AuNPs changes at a wavelength of 550–650 nm with a specificity of 15 × 10^5^ CFU/mL and 9 pg/µL by AuNP probes, with less detection time and cost-effective, rapid detection of bacteria [172]. In a study, recombinase polymerase amplification together with a fluorescent later flow immune assay containing Europium NPs was developed to detect *L. monocytogenes*, *Vibrio parahaemolyticus*, and *E. coli* O157:H7 quantitatively and showed a detection limit of the assay as 9 CFU/mL, 7 CFU/mL, and 4 CFU/mL, respectively, with high sensitivity and good specificity even at low concentrations of food-borne pathogens [173]. Antibiotic-functionalized (vancomycin-capped magnetic beads) centri-chronoamperometry with AuNPs was developed to detect Shigella flexneri and *B. cereus*, foodborne bacterial strains, at a concentration of at least 12 cells/mL and 3 cells/mL, respectively. Additionally, for the rapid detection of foodborne pathogens, vancomycin capped over the gold NPs surface effectively interacts with the bacterial membrane and is used for bacterial separation from the inoculated medium [174]. Dual-mode aptasensors against DNA fragments (cDNA) of *P. aeruginosa* were developed using two different-sized gold NPs (AuNPs). The 30 nm AuNPs carrying the aptamer were used as color signal probes, while the cDNA-15 nm AuNPs were used as SERS signaling probes. Without the target DNA, the two probes formed a duplex structure, while in the presence of the target DNA, the duplex structure was dissociated to link with the target DNA and reacted with TMB and hydrogen peroxide to produce a green color. The analyses were tested on various samples, including tap water and chicken meat samples, to validate them by detecting the levels of *P. aeruginosa* [175]. A novel technique was developed using trimethyl chitosan NPs to raise antibodies, which were further used to detect chitosan in fungal cell walls in various samples. The specificity varied among strains due to changes in the proportion of chitin content. The highest affinity was observed in *F. oxysporum*, while the least affinity was detected in *Trichoderma reesei*; thus, this method can be used for food quality control, human fungal infection, crop protection, etc. [176].

Unamplified genomic DNA (gDNA) nanosensors were developed using AuNPs capped with dextrin. A single-stranded DNA probe (ssDNAp) was used to stabilize dextrin-AuNPs and, in the presence of genomic ssDNA, an ssDNAp-target gDNA complex was formed, which increased the stability of the nanoparticle even in elevated ionic environments. This method effectively detected as little as 2.94 fM of pathogen DNA, rapidly exhibiting high specificity and selectivity [177]. A study engineered the receptor-binding protein exposed to phage M13, which could bind to the desired bacteria naturally. Engineered phage thiolation involves AuNP binding, which in turn causes phage aggregation and performs as a signal amplifier, causing color changes, which helps in the rapid detection of various species with high sensitivity and selectivity [178]. Nanomaterial-based biosensors applied in food science and food nanotechnology are presented in Supplementary Table S2.

### 3.9. Nanotechnology in Food Allergen Detection

Food allergies are one of the most serious public health issues for people who develop immunological reactions after eating certain foods. For many decades, the food industry has made numerous efforts to provide safe food to sensitive or allergic consumers [179]. Food allergens can be due to specific compounds like proteins in food or other ingredients like chemical compounds that are recognized by immune cells that trigger an immune reaction, leading to symptoms ranging from mild to life-threatening (Table 3) [180]. An electrochemical immunosensor was developed using a transducer consisting of an AuNP-coated carbon electrode to detect Ara h1, which is a major peanut allergen. This nanoimmunosensor quantifies Ara h1 from 12.6 to 2000 ng/mL, with 3.8 ng/mL as the detection limit, and it can be used for the analysis of cookies and chocolate [181]. In a study of allergen DNA target presence, a hybridizing chain reaction was increased to produce long dsDNA, which might not be adsorbed on AuNP surfaces, and in the presence of increased salt ions, the AuNPs became aggregated, leading to decreased UV absorption. In the absence of target DNA, hybridization was inhibited, which led to the decreased aggregation of AuNPs, and this technique had a detection limit of 0.5 nM with strong specificity [182]. The shellfish allergen tropomyosin was detected using a biosensor comprising chiral assemblies of trimer gold nanoparticles. The trimer AuNPs sensor is more selective for the determination of tropomyosin in the range of 0.1–15 ng mL^−1^. Thus, the use of trimer AuNPs is a simple, rapid, and accurate method for the detection of any allergen from shellfish [183]. A trichromatic xLFIA multiplex lateral flow immunoassay was developed using AgNPs and spherical and desert rose-like AuNPs. Antibodies developed using hazelnut allergenic and ovalbumin proteins were interfused on the metal NPs separately to introduce colored probes specifically for each protein. It was able to detect allergenic proteins as low as 0.1 mg/L and could be easily identified [184]. To detect peanut allergens (Ara h1) in bar chocolates, three different approaches were studied as follows: secondary antibody sandwich, label-free, and nanobead-enhanced assays. Comparatively, functionalized nanobeads can detect Ara h1 efficiently with a magnitude of two orders from 9 to 0.09 μg/mL and are suggested as a rapid method for detecting allergens in a very short period of time [185].

Au and Ag nanourchins with excellent SERS effects were developed and functionalized with modified β-lactoglobulin aptamers to detect the allergen β-lactoglobulin. In the presence of allergens, aptamer binding to allergens induced the dissociation of the Raman reporter molecule cDNA from Au-Ag nanourchins, which reduced the SERS signal. It provided a wide range of detection from 10 to 1000 ng/mL, finally settling at 0.07 ng/mL as the detection limit, and was considered a subtle and reliable detection method against allergens [186]. Kim et al. [187] developed a detection method using AuNPs against peanut allergen (Ara h1) in a cookie sample. Ara h1 detection (0.19 mg) was based on the aggregation of switchable linkers and AuNPs per 30 g of cookie at a detection time of 30 min and was suggested to be used for a wide range of allergens. The cell sensor was developed using hydrazide-functionalized multiwalled carbon nanotubes and flower-like copper oxide NPs, and 3D printing technology was used to print duodenal microvillus shapes on the electrode screen to detect the wheat allergen gliadin. Bioprinted cell sensors with bioprints delicately sense 0.1–0.8 ng/mL as the linear detection range and 0.036 ng/mL as the detection limit possessing excellent stability and reproducibility, which could be used in the application of food safety detection and evaluation [188]. An immunoassay method was developed for the quantitation of arginine kinase (AK) (an allergen in shrimp food samples) using AuNPs and quantum dots. AuNPs were modified with antibody 1 against AK, and a secondary antibody was added to the quantum dots. In the presence of AK, FRET would occur between nanoparticles and quantum dots, causing a decreased signal, which was correlated linearly with the AK concentration in the range of 1.0 × 10^−6^–1.0 × 10^−3^ mg/mL with a detection limit of 0.11 ng/mL [189]. A casein-detecting sensor was developed using a molecularly imprinted polymer nanoparticle and incorporated into a label-free surface plasmon resonance (SPR)-based sensor that showed high affinity towards bovine α-casein with a detection limit of 127 ± 97.6 ng mL^−1^, which is rapid and cost-effective compared to commercially available ELISA kits [190]. Fish allergen parvalbumin was detected using a rapid and highly sensitive sandwich immunochromatographic assay using two different primary monoclonal antibodies: one as a capture antibody, and the other conjugated with Fe_3_O_4_/Au NPs (a detection reagent). The color developed was positively correlated with the concentration of parvalbumin, with a detection limit of 0.691 ng/mL and 2 ng/mL, qualitatively and quantitatively, with a detection time of 15 min [191]. A sandwich enzyme-linked immunosorbent assay (sELISA) was developed to detect bovine β-lactoglobulin using CdTe quantum dots, which were functionalized using catalase-mediated fluorescence quenching in the presence of signal output H_2_O_2_. It showed high specificity and sensitivity with an H_2_ detection limit of 0.49 ng mL^−1^ and was superior to commercially available ELISA kits [192]. Bovine serum albumin was detected using a polydiacetylene-triblock copolymer nanosensor in the samples of milk and dairy products to discriminate between native and denatured proteins based on the blue-to-red color transition and was reported as a low-cost sensor for the rapid detection of BSA [193].

**Table 3:** Nanotechnology for allergen detection.

S.No	Nanoparticle	Food Allergen	Detection Method	References
1.	AuNPs	Pollen allergens	DNA hairpin self-assembly Salt-induced non-cross-linking aggregation of gold NPs densely modified with short DNA Detection limit: 0.2 ng/mL (130-fold greater)	[194]
2.	AuNPs	Fish products allergen parvalbumin	Capillary immunochromatographic assay Visual detection limit: 70 ng/mL Semi-quantitative limit of detection: 40 ng/mL	[195]
3.	AuNPs	Soybean allergen β-conglycinin.	Sandwich lateral flow immunochromatographic detection strips; detection limit: 1 μg/mL.	[196]
4.	Immunomagnetic NPs	Peanut allergen Ara h2	Lateral flow assay; visual detection limit of 0.1 mg/kg; high reproducibility	[197]
5.	platinum NPs	Cow milk allergy β-lactoglobulin	Sandwich enzyme-linked immunosorbent assay Detection limit 0.12 ng/mL	[192]
6.	Quantum dots	Cow milk allergy β-lactoglobulin	Sensitive fluorescent sandwich enzyme-linked immunosorbent assay; limit of detection was 0.49 ng mL^−1^	[198]
7.	AuNPs	Gluten	An impedimetric biosensor Limit of detection of 0.05 mg L^−1^	[199]
8.	AuNPs polyethyleneimine-multiwalled carbon nanotubes nanocomposite	Kidney bean lectin	Label-free voltammetric immunosensor Detection limit of 0.023 μg/mL Rapid quantification	[200]
9.	immunomagnetic nanoparticle	Peanut allergen Ara h2	Lateral flow assay Visual limit of detection 0.01 μg/mL	[197]
10.	CdSe/ZnS quantum dots	Dairy product α-lactalbumin	Competitive fluorescence-linked immunosorbent assay Detection ranging from 0.1 to 1000 ng/mL LOD at 0.1 ng/mL	[201]

### 3.10. Nanotechnology for Inhibition of Microbial Growth and Biofilm Formation

**Figure 4:**
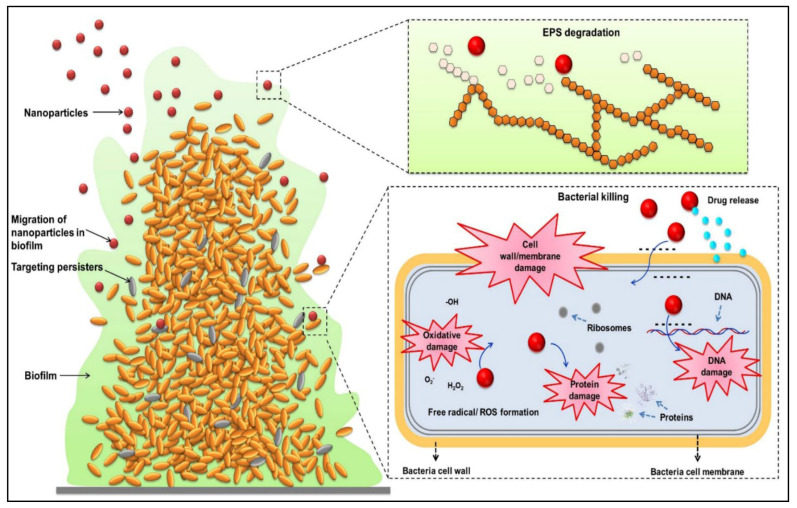
Role of nanotechnology in eradicating biofilm formed by pathogenic bacteria, adopted from [218].

Bacteria form biofilms, which aid in adhering to surfaces and assisting in the maturation and dispersion of bacteria, both of which are required for the spread of bacteria and the creation of contamination [202]. Green synthesized Ag and ZnO NPs are the most explored nanomaterials studied for the prevention of bacterial growth and the production of biofilms. It has been reported that AgNPs synthesized by green methods using different parts of plant extracts significantly inhibit the growth of *E. coli*, *P. aeruginosa*, *S. aurous*, *B. cereus,* and *K. pneumonia*, and also inhibit biofilm formation [203,204,205]. In another study, it was found that biologically synthesized ZnO NPs enhanced the antimicrobial activity and inhibited the biofilm formation by *E. coli* and *S. aureus* [206,207]. *L. monocytogenes* is a bacterium that causes listeriosis, a foodborne disease that increases the risk of forming biofilms in the food industry and helps the bacteria to survive in challenging environments. Thus, developing antibiofilm materials for food-processing environments is highly necessary [208]. Nanomaterials with visible light illumination inactivate and reduce the biofilm of *L. monocytogenes* on TiO_2_-coated aluminum and stainless steel plates after 72-hand illumination, which is suggested as a safe and effective method for use in facilities like food processing for biofilm inhibition [208]. The combination of Au, Ag, and chitosan NPs at higher concentrations effectively inhibited biofilm growth and decreased metabolic activity (80%) in all the tested strains except for *Salmonella enterica infantis*; however, nanoparticles were not able to completely remove biofilm mass [209]. Biogenic AgNPs were developed from *Convolvulus arvensis* extract and also with chitosan to test against Gram-positive and Gram-negative bacteria. It was reported that nanoparticles decreased the bacterial cell count (*S. aureus* and *E. coli*) and also exerted antibiofilm potential against *P. aeruginosa*. Additionally, nanoparticles showed an anticancer effect by increasing cytotoxicity against breast cancer cell lines and altogether exerting an antibacterial, anti-biofilm, and anticancer effect [210]. A nanodrug delivery system was developed using chitosan NPs, phosphatidylcholine, and gentamicin and showed that it could effectively damage and remove the biofilm formed by pathogens via permeation through the biofilm by NPs that were engulfed by macrophages, which led to the killing of bacteria, suggesting that NPs could be a promising nano-antibacterial agent against biofilms and intracellular bacteria [211]. AgNPs were coated onto stainless steel and a polyethylene surface to test the influence of biofilm formation, which enhances the contact of microorganisms [212]. AgNP-decorated amphiphilic carbonaceous particles were developed and incorporated into urinary catheters. It improved the mechanical properties and surface wettability; possessed bacterial resistance, adhesion, and biofilm formation without inducing toxicity in rabbits; and acted as an effective antibacterial and antibiofilm agent [213]. Usnic acid-loaded chitosan NPs were prepared via simple ionic gelification and showed 24% encapsulation and 5.2% loading capacity, with a spherical and rough surface. It was found that usnic acid-chitosan NPs exerted significant antibacterial activity against persister cells and inhibited biofilm formation by Gram-negative and Gram-positive bacteria. Thus, it could be an effective alternative method for the use of usnic acid in NPs [214]. AgNPs prepared from the shoot extract of *Tamarix nilotica* showed that AgNPs significantly decreased the swimming, motility, cell growth, and biofilm formation of *Listeria* test strains. Ultimately, NPs exerted antibiofilm and wound-healing properties and could be effectively used to prevent infection with *L. monocytogenes* in the food industry [215]. ZnO NPs were tested against *Campylobacter jejuni* biofilms and it was found that NPs penetrated the biofilm within 1 h without damaging it and directly interacted with sessile cells in biofilms. NPs then induce increased free radical generation and alter DNA/RNA bases, leading to *C. jejuni* cell death [216]. Clove oil-loaded chitosan NPs and gelatin electrospun nanofibers were tested against biofilms of *E. coli*, and it was found that nanofiber treatment effectively reduced biofilm formation and maintained the color and flavor of cucumber for more than 4 days [217]. The role of nanotechnology in eradicating biofilms formed by pathogenic bacteria is shown in Figure 4.

## 4. Limitation, Challenges, and Future Prospectus 

Nanotechnology is essential for achieving food security and can enhance crop production by controlling microbial, pest, and weed diseases with high nutritional value, security, and safety. Nanotechnology helps in the development of materials, devices, or systems at the nanometer scale and possesses a high surface-to-volume ratio with unique physiochemical properties in the agricultural sector, as reviewed above. Nanotechnology has a significant impact on the food industry because it allows for the superior processing, packaging, and long-term storage of food. This has led to an enormous increase in the food industry by improving both the flavor and texture of food. Via pathogen detection, nanomaterials and nanosensors improve security by assisting consumers and providing glimpses into the overall condition of the food inside, as well as its nutritional status. Since the majority of food bioactives that fight different diseases are hydrophobic in nature and have low bioavailability and stability, nanotechnology-based delivery methods have been able to enhance food bioactive compound bioavailability and targeted delivery [219]. Despite the many innovative products and development processes in the food industry, nanotechnology faces a significant challenge in producing edible and non-toxic nanocompounds or delivery systems that are unsafe for human consumption using cost-effective methods [220]. It has been reported that the bioaccumulation of nanomaterials, such as AgNPs derived from nanopackaging, has been observed in both food and humans [221]. As a result of their release from contaminated food or into the environment, the widespread use of nanomaterials in food packaging may be cause for concern. NPs can enter the body via ingestion, inhalation, or skin contact, and due to their nanoscale size and large surface area, they are capable of readily penetrating cells and causing toxicity in distant areas of the body [222]. At higher concentrations, NPs can cause toxicity by denaturing the body’s proteins and enzymes due to an increase in the production of free radicals, which is considered a key mechanism leading to oxidative stress in humans [223,224]. According to a recent study, inflammation, oxidative stress, genotoxicity, chromosome instability, mitochondrial damage, and apoptosis have been shown to be caused by TiO_2,_ which is used as a food additive or in another form of food upon entering the body [225]. However, data related to the dispersion of nanoparticles from packaging materials into food and their ultimate toxicological effects are limited [226]. Moreover, the use of nanopesticides and nanofertilizers in the agriculture sector poses a serious health risk to farmers since they can contaminate water, soil, or the atmosphere. It is expected that once NPs accumulate in the soil, plant growth will be hampered, and they may accumulate in edible tissues as well [227]. As soon as the NPs are released into the agro-environment, they undergo a variety of transformations that may cause toxicity in both plants and humans [226]. Due to the inherent risk to human health posed by the use of NPs as pesticides and microbicides, strict adherence to risk assessment procedures is required when preparing food [228]. According to reports, exposure to AgNPs causes phytotoxicity in aquatic plants like *Egeria densa* and *Juncus effusus* by increasing lipid peroxidation, changing physiological traits, and causing additional stress in submerged macrophytes [229]. It has been proposed that green nanofillers are needed for nanocomposite research to ensure the safety of the environment, plants, humans, animals, and aquatic life [226]. For a safer implementation of NPs in the food sector, effective regulations, policies, and regulatory laws are required. For instance, it is important to educate the public on the effects of nanomaterials on human health, safety, and the environment. Additionally, risk analysis and safety requirements must be met before nanofood is made available in the market [230]. According to European Union regulations, any nanotechnology-based food constituent, such as nanofoods or food packaging, is required to pass a Food and Drug Administration (FDA) safety assessment before being approved for use [231]. However, many countries do not follow proper regulations for the production and processing of nanomaterials, so toxicological screening is critical for the application of nanotechnology [219]. Apart from nanotoxicity, there are practical limitations to developing nanocoating strategies to regulate the surface properties of substrates for application in the environment, biosystems, food systems, etc. The coating of fruits is time-consuming and the desired coating is not achievable. Thus, rapid and novel coating strategies are highly demanded [91]). Upon genetic editing of plants, NP-loaded cargo size and amount for CRISPR DNA and protein cargoes have been well established. Additionally, it is not clearly understood whether NPs can carry CRISPR reagents to plant mitochondria for genome modification. It remains unknown whether these NPs can carry CRISPR reagents to plant plastids and mitochondria for genome modification [30].

## 5. Conclusions

Recently, nanotechnology has provided a path for increasing food availability and developing new agricultural products, particularly by minimizing nutrient losses from fertilizers and improving food yield through pest and nutrient management. Nanofertilizer improved seedling vigor index, increased reserve food-mobilizing enzyme activities, increased antioxidant enzymes, decreased lipid peroxidation, stimulated chlorophyll content, and effectively enhanced crop yield by promoting source activity. Nanotechnology helps in food processing, food modification, stability, sensing, enhanced shelf life, and decreased food losses. Nanomaterials protect plants against pathogens by inhibiting microbial growth, inducing innate plant immunity, and delivering pesticides and micronutrients. Furthermore, NPs improve stress tolerance by reducing abscisic acid, regulating gibberellic acid, and improving seed germination. Nanoencapsulation helps regulate and release the active ingredients; protects them from chemicals, heat, and moisture; and enhances thermal stability and photostability. Nanotechnology-based foods challenge both the private sector and the government, assuring confidence in consumers and accepting nanofoods in the marketplace. In recent years, it has been extensively reported that nanocolloidal particles are actively utilized in various facets of the food industry, including the safety and quality of food, nutrition, processing, and packaging. The structure and function of colloidal particles are vital for the development of foods that are safer, more nutritious, of higher quality, and more sustainable. The use of NPs in food packaging has fewer health risks than their use as ingredients in food. During the process of manufacturing and application, nanomaterials pose a constant risk of entering the food chain via water, air, and soil. This can result in DNA damage, disruption of cell membranes, and cell death. Furthermore, in vivo research on the effects of nanofoods on human and animal health has been extremely limited. Therefore, it is highly recommended to conduct increasing in vivo research on the effects of nanofoods to determine their potential hazards at the molecular and genetic levels. Additionally, future investigations could also control the potential hazards posed by nanoparticles or nanocomposites via the application of sustainable synthesis techniques and searching for simple, rapid, ecofriendly, and inexpensive strategies for the breakdown and removal of existing nanomaterials at attack sites. Furthermore, many nations lack the appropriate rules and laws for the manufacture and handling of nanomaterials. Therefore, comprehensive federal laws and regulations, along with a strict toxicological assessment system, are urgently required for the legitimate utilization of nanotechnology in the food manufacturing industry. Thus, the use of the aforementioned nanotechnologies could significantly enhance the production and processing of foods and the quality of products, which will improve human health and well-being if handled and governed effectively.

## Figures and Tables

**Table 2:** Application of nanotechnology in food packaging.

S.No.	Nanomaterials	Food Item Packed/Uses	Function	References
1.	Silver ion and silver NPs	Meats (tuna, ham, and turkey)	Silver ions and NPs release from food touch paper in food stimulants; antibacterial capacity of released silver in turkey against Gram-negative bacteria.	[133]
2.	AgNPs	Chicken meat	Absence of silver in chicken meatballs; analysis of microbiological tests showed no difference between meatballs stored in silver-nanoparticle plastic bags and control bags.	[134]
3.	Chitosan and ZnO NPs loaded gallic-acid films	For Active food packaging	Films possess a significant antibacterial potential and strong antioxidant behavior compared to pristine chitosan.	[135]
4.	Nanocrystals from cellulose	Aerogel absorbers for food packaging	A porous and uniform structure with a water absorption capacity.	[136]
5.	Graphite carbon nitride nanosheets/MoS_2_ nanodots into konjac glucomannan (KGM) matrix	Cherry tomatoes	Films have broad-spectrum antibacterial activity and are safe to use.	[137]
6.	Stable silver NPs using chitosan	Meat	Effective antimicrobial activity against Gram-positive and Gram-negative bacteria; increased bio-activity and extended the meat shelf-life by one week.	[138]
7.	Carboxymethyl cellulose/cellulose nanocrystals immobilized Ag NPs	Strawberries	Antibacterial activities against *E. coli* and *S. aureus*; extended shelf life of strawberries up to 7 days; increased tensile strength and reduction in air permeability.	[139]
8.	Nanosilver-coated low-density polyethylene	Active food packaging	Under oven heating, a significant reduction in silver migration and antimicrobial activity.	[140]
9.	Polylactic acid/titanium dioxide/lycopene nanocomposite film	Margarine	Films act as visual indicators (red to yellow) of the oxidation variations during the storage of packaged margarine; increased shelf life of margarine; controlled oxidative factors.	[141]
10.	AgNPs using mango peel extract film	Strawberries	Improved the mechanical properties of the film and its barrier to water vapor and oxygen; exhibited excellent antibacterial properties against *E. coli* and *S. aureus*; improved the shelf life of strawberries.	[142]
11.	Chitosan NPs	Active bioplastic packaging	Antibacterial activity against the growth of *E. coli* and effective antioxidant potential	[143]
12.	Chitosan/grape seed extract/silver nanoparticle composite	Grapes	Antimicrobial and antioxidant activities; reduced decay percentage and weight loss; inhibited the total mold count during storage; extended shelf life of grapes.	[144]

## Data Availability

Not applicable.

## Supplementary Materials

The following supporting information can be downloaded at: https://www.mdpi.com/article/10.3390/plants12132565/s1, Supplementary Table S1: Nanoparticles employed for the detection of foodborne pathogens; Supplementary Table S2: Nanomaterial-based biosensors applied in food science and food nanotechnology. References [232,233,234,235,236,237,238,239,240,241,242,243,244,245,246,247,248,249,250,251,252,253,254,255,256,257,258,259,260,261,262,263,264,265,266,267,268,269,270,271,272,273,274] are also cited in Supplementary Materials.
