# Supplementary material for: Nanotechnology in Food and Plant Science: Challenges and Future Prospects
Source: Plants (Basel). 2023 Jul 6;12(13):2565. doi: 10.3390/plants12132565 (PMC10346217; doi:10.3390/plants12132565)
Supplement: Supplementary file 1 [file plants-12-02565-s001.zip › plants-2464530-supplementary.pdf]

Supplementary Table S1. nanoparticles employed for the detection of foodborne pathogens

Nanoparticles	Pathogens	Detection limit	Ref
Magnetic bead/quantum dot	*E. coli* 0157:H7	10^3 CFU/mL	232
Magnetic nanoparticle	*E. coli* O157:H7, *S. aureus*, *S. epidermidis*	10^4 CFU/mL, 8 CFU/mL, 10^3 CFU/mL	233
Immunomagnetic liposome nanoparticle	*Cronobacter sakazakii*	10^3 CFU/mL	234
RuBpy doped silica	*E. coli* 0157:H7	1 cell/mL	232,235
Liposome nanoparticles	*Salmonella typhimurium*	10^2 CFU/mL	236
Aptamer conjugated gold nanoparticles	*S. typhimurium*	10^4 CFU/mL	237,238
Quantum dot	*Salmonella enterica* serotype *Typhi*, *E. coli* O157:H7, *Listeria monocytogenes*	103–106 cells/ml	239-242
Single walled carbon nanotube	*E. coli*	NA	233
Gold/silicon nanorod	*Salmonella enterica serotype Typhi*; Respiratory syncytial virus	Not reported	243
Gold nanorod	*E. coli* O157:H7	1-10 CFU/mL	244
Gold nanoparticle	*Salmonella enterica serotype Typhi*	98.9 CFU/mL	245

Supplementary Table S2. Nanomaterial-based biosensors applied in food science and food nanotechnology

Sensor type	Nanomaterial	Sample	Analyte	Function	Ref.
Electrochemical	CuO nanostructure	Cabbage and spinach extract	Residual pesticides (chlorpyrifos, fenthion and methyl parathion)	Surface area enhancement	246
	AuNPs label	Skimmed milk	Pathogens (*Salmonella*)	Electrochemical signal generation	247
	AuNPs electrodeposited on GCE	Edible oils	Antioxidants ((BHA), (BHT) and (TBHQ)	Improved conductivity due to surface area enlargement and increase of active sites	248
	Cd QDs encapsulated in ZIF-8 metal organic framework	Milk	Pathogens (*E. coli* O157:H7)	Electrooctochemical signal generation due to Cd etching to Cd(II)	249
	TiO_2/CeO_2 NPs	*In vivo* rat brain	Adulterants (dopamine)	Enhanced sensitivity	250
	Graphene nano ribbons (GNR)	Fruit juice	Antioxidants (ascorbic acid)	Enhanced surface area and electrochemical properties	251
	Graphene-Cu NPs	Banana and bovine milk	Mannitol, sucrose, lactose, glucose, and fructose	Oxidation of carbohydrate	252
		—	Mannitol, sucrose, glucose, and fructose	CNTS provided high surface area. Cu NPs enabled oxidation of carbohydrate	253
	PtNPs	Soil and water	Residual pesticides (paraoxon)	Surface area, conductivity and enzyme loading enhancement	254

	AuNPs loaded on MWCNT	Milk	Toxins (BPA)	Enhanced surface area and conductivity	255
	MWCNT	Baby feeding bottles	Toxins (BPA)	Conductive and functionalizable layer	256
	-GNRs/GO-Pd/Au core–shell nanocrystallines (Pd/Au CSNs)<br><br>-CdSe@CdS QDs	Tomato sauce<br>Chili sauce<br>Chili powder	Adulterants, Sudan I	GO provided a matrix for antibody immobilization. GNRs/ GO enhanced the surface area thus enhanced the signal generation<br>Pd/Au CSNs provides catalytic activity and high surface area	257
	GODs-AuNPs	—	Heavy metals (Hg^{2+}, Cu^{2+})	Preconcentration	258
	GSH@Fe_3O_4	Water	Heavy metals (Pb^{2+}, Cd^{2+})	Preconcentration	259
	SWCNTs gold	Water	Heavy metals (Hg^{2+})	Provides a high surface area of thiophenol- modified SWCNTs gold electrode	260
Optical	CeO_2 NPs	Tea and medicinal mushroom	Antioxidants (ascorbic acid, gallic acid, vanillic acid, quercetin, caffeic acid, and epigallocatechin gallate)	Catalytic activity	261
	CeO_2 NPs	Aqueous media and serum	Adulterants (dopamine)	Colorimetric signal	262, 263

	CeO_2 NPs	Milk	Mycotoxin (ochratoxinA)	Catalytic activity	264
	AuNPs	Milk powder	Adulterants (melamine)	SPR optical properties	265
	Au nanocolloid	Tomato sauce and chili powder	Adulterants, Sudan I	Visible colorimetric signal on nitrocellulose strip	266
	Au nanocage	Green tea	Antioxidants (gallic acid)	Morphological change induces LSPR change upon seed-mediated growth of Ag	267
	MnO_2 nanosheets	Orange and orange juice	Antioxidants (ascorbic acid)	Catalytic activity	268
	Nitrogen-doped carbon dots	—	Heavy metals (Hg^{2+}, Cu^{2+}, Fe^{3+})	Fluorescent response	269
	DNA–AuNPs	—	Heavy metals (Hg^{2+})	Colorimetric response due to aggregation	270
	AuNPs	Cucumber and hamburger extracts	Pathogens (*E. coli* and *Salmonella* sp.)	SPR optical properties	271
	Au@AgNPs	Milk	Antibiotics (kanamycin)	SERS activity	272
	AuNR $MnFe_2O_4$@Au	—	Pathogens (*S. aureus*)	SERS activity Separation	273
	AuNPs	Honey	Antibiotics (26 sulfonamides)	—	274

References

[232] Yang L, Li Y. Quantum dot bioconjugates for simultaneous detection of Escherichia coli O157:H7 and Salmonella Typhimurium. Analyst 2006;131:394e401.
[233] Zhao X, Hilliard L, Mechery S, Wang Y, Bagwe R, Jin S. A rapid bioassay for single bacterial cell quantitation using bioconjugated nanoparticles. Proc Natl Acad Sci USA. 2004;42:15027e32.
[234] Shukla S, Lee G, Song X, Park S, Kim M. Immunoliposomebased immunomagnetic concentration and separation assay for rapid detection of Cronobacter sakazakii. Biosens Bioelectron 2016;77:986e94.
[235] Su SL, Li Y. Quantum dot biolabeling coupled with immunomagnetic separation for detection of Escherichia coli O157:H7. Anal Chem 2004;76(16):4806e10.
[236] Zhou L, Lv S, He G, He Q, Shi BI. Effect of PE/AG2O nanopackaging on the quality of apple slices. J Food Qual 2011;34(3):171e6.
[237] Oh SY, Heo NS, Shukla S, Cho HY, Ezhil Vilian AT, Kim J et al. Development of gold nanoparticle aptamer-based LSPR sensing chips for the rapid detection of Salmonella typhimurium in pork meat. Sci Rep 2017;7:e10130.
[238] De Souza Reboucas J, Esparza I, Ferrer M, Sanz ML, Irache JM, Gamazo C. Nanoparticulate adjuvants and delivery systems for allergen immunotherapy. J Biomed Biotechnol 2012:e474605.
[239] Hahn MA, Keng PC, Krauss TD. Flow cytometric analysis to detect pathogens in bacterial cell mixtures using semiconductor quantum dots. Anal Chem 2008;3:864e72.
[240] Yang L, Li Y. Quantum dots as fluorescent labels for quantitative detection of S. typhimurium in chicken carcass wash water. J Food Protect 2005;6:1241e5.
[241] Tully E, Hearty S, Leonard P, O'Kennedy R. The development of rapid fluorescence-based immunoassays, using quantum dot-labelled antibodies for the detection of L. monocytogenes cell surface proteins. Int J Biol Macromol 2006;1e3:127e34.
[242] Wang C, Irudayaraj J. Gold nanorod probes for the detection of multiple pathogens. Small 2008;12:2204e8.
[243] Dungchai W, Siangproh W, Chaicumpa W, Tongtawe P, Chailapakul O. S. typhi determination using voltametric amplification of nanoparticles: a highly sensitive strategy for metalloimmunoassay based on a copper-enhanced gold label. Talanta 2008;2:727e32.
[244] Fu J, Park B, Siragusa G, Jones L, Tripp R, Zhao Y. An Au/Si hetero-nanorod-based biosensor for Salmonella detection. Nanotechnology 2008;15:e155502.
[245] Vikesland PJ, Wigginton KR. Nanomaterial enabled biosensors for pathogen monitoring e a review. Environ Sci Technol 2010;10:3656e69.
[246] M. M. Tunesi, et al., Functionalised CuO nanostructures for the detection of organophosphorus pesticides: a non- enzymatic inhibition approach coupled with nano-scale electrode engineering to improve electrode sensitivity, Sens. Actuators, B, 2018, 260, 480–489.
[247] A. S. Afonso, et al., Electrochemical detection of Salmonella using gold nanoparticles, Biosens. Bioelectron., 2013, 40(1), 121–126.

[248] X. Lin, Y. Ni and S. Kokot, Glassy carbon electrodes modified with gold nanoparticles for the simultaneous determination of three food antioxidants, Anal. Chim. Acta, 2013, 765, 54–62.

[249] M. Zhong, et al., An electrochemical immunobiosensor for ultrasensitive detection of Escherichia coli O157:H7 using CdS quantum dots-encapsulated metal-organic frameworks as signal-amplifying tags, Biosens. Bioelectron., 2019, 126, 493–500.

[250] J. Njagi, et al., Amperometric detection of dopamine in vivo with an enzyme based carbon fiber microbiosensor, Anal. Chem., 2010, 82(3), 989–996.

[251] Y. Yang, et al., Electrochemical evaluation of total antioxidant capacities in fruit juice based on the guanine/ graphene nanoribbon/glassy carbon electrode, Talanta, 2013, 106, 206–211.

[252] Q. Chen, L. Zhang and G. Chen, Facile preparation of graphene-copper nanoparticle composite by in situ chemical reduction for electrochemical sensing of carbohydrates, Anal. Chem., 2012, 84(1), 171–178.

[253] Y. Fu, L. Zhang and G. Chen, Preparation of a carbon nanotube-copper nanoparticle hybrid by chemical reduction for use in the electrochemical sensing of carbohydrates, Carbon, 2012, 50(7), 2563–2570.

[254] J. A. Hondred, et al., Printed graphene electrochemical biosensors fabricated by inkjet maskless lithography for rapid and sensitive detection of organophosphates, ACS Appl. Mater. Interfaces, 2018, 10(13), 11125–11134.

[255] N. B. Messaoud, et al., Electrochemical sensor based on multiwalled carbon nanotube and gold nanoparticle modified electrode for the sensitive detection of bisphenol A, Sens. Actuators, B, 2017, 253, 513–522.

[256] T. Anirudhan, V. Athira and V. C. Sekhar, Electrochemical sensing and nano molar level detection of Bisphenol-A with molecularly imprinted polymer tailored on multiwalled carbon nanotubes, Polymer, 2018, 146, 312–320.

[257] C. Wang, et al., Multiple signal amplification electrochemiluminescent immunoassay for Sudan I using gold nanorods functionalized graphene oxide and palladium/aurum core-shell nanocrystallines as labels, Electrochim. Acta, 2018, 278, 352–362.

[258] S. L. Ting, et al., Graphene quantum dots functionalized gold nanoparticles for sensitive electrochemical detection of heavy metal ions, Electrochim. Acta, 2015, 172, 7–11.

[259] M. Baghayeri, et al., A simple approach for simultaneous detection of cadmium (II) and lead (II) based on glutathione coated magnetic nanoparticles as a highly selective electrochemical probe, Sens. Actuators, B, 2018, 273, 1442–1450.

[260] J. Wei, et al., Stripping voltammetric determination of mercury (II) based on SWCNT-PhSH modified gold electrode, Sens. Actuators, B, 2014, 190, 968–974.

[261] E. Sharpe, et al., Portable ceria nanoparticle-based assay for rapid detection of food antioxidants (NanoCerac), Analyst, 2013, 138(1), 249–262.

[262] A. Hayat, et al., Redox reactivity of cerium oxide nanoparticles against dopamine, J. Colloid Interface Sci., 2014, 418, 240–245.

[263] G. Bu¨lbu¨l, et al., Reactivity of nanoceria particles exposed to biologically relevant catechol-containing molecules, RSC Adv., 2016, 6(65), 60007–60014.

[264] G. Bu¨lbu¨l, A. Hayat and S. Andreescu, ssDNA-Functionalized Nanoceria: A Redox-Active Aptaswitch for Biomolecular Recognition, Adv. Healthcare Mater., 2016, 5(7), 822–828.

[265] S. Y. Oh, et al., Cuvette-Type LSPR Sensor for Highly Sensitive Detection of Melamine in Infant Formulas, Sensors, 2019, 19(18), 3839.

[266] J. Wang, et al., Nanocolloidal gold-based immuno-dip strip assay for rapid detection of Sudan red I in food samples, Food Chem., 2013, 136(3–4), 1478–1483.

[267] Y. Wang, et al., Morphological control of nanoprobe for colorimetric antioxidant detection, Biosens. Bioelectron., 2018, 122, 183–188.

[268] L. He, et al., Rapid and sensitive colorimetric detection of ascorbic acid in food based on the intrinsic oxidase-like activity of MnO2 nanosheets, Luminescence, 2018, 33(1), 145–152.

[269] Z. Wang, et al., Fluorescence sensor array based on amino acid derived carbon dots for pattern-based detection of toxic metal ions, Sens. Actuators, B, 2017, 241, 1324–1330.

[270] J. S. Lee, M. S. Han and C. A. Mirkin, Colorimetric detection of mercuric ion (Hg2+) in aqueous media using DNA-functionalized gold nanoparticles, Angew. Chem., Int. Ed., 2007, 46(22), 4093–4096.

[271] H. Vaisocherov´a-L´ısalov´a, et al., Low-fouling surface plasmon resonance biosensor for multi-step detection of foodborne bacterial pathogens in complex food samples, Biosens. Bioelectron., 2016, 80, 84–90.

[272] Y. Jiang, et al., Ultrasensitive analysis of kanamycin residue in milk by SERS-based aptasensor, Talanta, 2019, 197, 151– 158.

[273] J. Wang, et al., Facile synthesis of Au-coated magnetic nanoparticles and their application in bacteria detection via a SERS method, ACS Appl. Mater. Interfaces, 2016, 8(31), 19958–19967.

[274] Y. Chen, et al., Gold immunochromatographic sensor for the rapid detection of twenty-six sulfonamides in foods, Nano Res., 2017, 10(8), 2833–2844.
